# Bioinformatics analysis of immune characteristics in tumors with alternative carcinogenesis pathways induced by human papillomaviruses

**DOI:** 10.1186/s12985-023-02241-6

**Published:** 2023-12-04

**Authors:** Michal Smahel, Jaroslav Nunvar

**Affiliations:** Department of Genetics and Microbiology, Faculty of Science, Charles University, BIOCEV, 252 50 Vestec, Czech Republic

**Keywords:** Papillomavirus, Carcinogenesis, Immunotherapy, STING, LILR

## Abstract

**Background:**

Human papillomaviruses (HPVs) induce a subset of head and neck squamous cell carcinomas (HNSCC) and anogenital cancers, particularly cervical cancer (CC). The major viral proteins that contribute to tumorigenesis are the E6 and E7 oncoproteins, whose expression is usually enhanced after the integration of viral DNA into the host genome. Recently, an alternative tumorigenesis pathway has been suggested in approximately half of HNSCC and CC cases associated with HPV infection. This pathway is characterized by extrachromosomal HPV persistence and increased expression of the viral E2, E4, and E5 genes. The E6, E7, E5, and E2 proteins have been shown to modify the expression of numerous cellular immune-related genes. The antitumor immune response is a critical factor in the prognosis of HPV-driven cancers, and its characterization may contribute to the prediction and personalization of the increasingly used cancer immunotherapy.

**Methods:**

We analyzed the immune characteristics of HPV-dependent tumors and their association with carcinogenesis types. Transcriptomic HNSCC and CC datasets from The Cancer Genome Atlas were used for this analysis.

**Results:**

Clustering with immune-related genes resulted in two clusters of HPV16-positive squamous cell carcinomas in both tumor types: cluster 1 had higher activation of immune responses, including stimulation of the antigen processing and presentation pathway, which was associated with higher immune cell infiltration and better overall survival, and cluster 2 was characterized by keratinization. In CC, the distribution of tumor samples into clusters 1 and 2 did not depend on the level of E2/E5 expression, but in HNSCC, most E2/E5-high tumors were localized in cluster 1 and E2/E5-low tumors in cluster 2. Further analysis did not reveal any association between the E2/E5 levels and the expression of immune-related genes.

**Conclusions:**

Our results suggest that while the detection of immune responses associated with preserved expression of genes encoding components of antigen processing and presentation machinery in HPV-driven tumors may be markers of better prognosis and an important factor in therapy selection, the type of carcinogenesis does not seem to play a decisive role in the induction of antitumor immunity.

**Supplementary Information:**

The online version contains supplementary material available at 10.1186/s12985-023-02241-6.

## Background

Human papillomaviruses (HPVs) are associated with approximately 4% of human cancers worldwide [1]. They are involved in nearly all cases of cervical carcinoma (CC) and up to 35% of head and neck squamous cell carcinoma (HNSCC) [2]. The incidence of HPV-induced cancers is still increasing, particularly oropharyngeal cancers [3, 4]. A significant reduction in HPV-driven carcinomas due to preventive vaccination is expected after 2050 [5].

Of the more than 200 HPV types identified, approximately 15 types are considered to be high-risk with respect to malignant tumor development [6]. The viral proteins E6 and E7 are the major viral oncoproteins, but the oncogenic potential of the E5 protein has also been recognized [7]. These viral oncoproteins interact with numerous cellular proteins and influence various cancer hallmarks [7, 8]. Integration of the HPV genome into the host DNA is considered an important step in carcinogenesis, where the viral E2 gene is usually disrupted and the expression of the E6 and E7 oncogenes is enhanced [9]. In addition, the expression of cellular genes in the vicinity of an integration site may be altered, thereby supporting carcinogenesis [10].

Ren et al. characterized an alternative HPV carcinogenesis pathway that is not dependent on E6/E7 expression and viral genome integration but is driven by episomal expression of the E2, E4, and E5 genes [11]. They identified this subtype of carcinogenesis in approximately half of HPV-positive cervical and pharyngeal cancers, demonstrated activation of fibroblast growth factor receptor signaling, and verified alternative carcinogenesis in in vitro and in vivo models. The effect of the E2/E4/E5 pathway on cell proliferation and survival was p53 dependent, which may be mediated by E2 binding [12, 13]. In HNSCC, patients with E2/E4/E5 carcinogenesis had a slightly worse prognosis than those with E6/E7 carcinogenesis, but the difference was not significant.

Since immunotherapy is increasingly used against cancer, the immunological characteristics of tumors are being studied to identify prognostic and predictive biomarkers and therapeutic targets. HPV proteins affect both innate and adaptive immune responses and contribute to tumor escape from host immunity [7, 14]. Therefore, the different levels of HPV oncoproteins associated with the two alternative pathways of carcinogenesis may provide a basis for different therapeutic targets and responses to immunotherapy. To reveal possible immunological variance between tumors with high or low E2/E4/E5 expression, we performed bioinformatics analysis of transcriptomic datasets of HNSCC and CC samples.

## Materials and methods

### Sample selection

CC and HNSCC tumor samples from The Cancer Genome Atlas (TCGA Research Network: https://www.cancer.gov/tcga) were evaluated based on their expression of HPV genes as determined by Ren et al. [11]. A total of 138 and 54 CC and HNSCC samples, respectively, with robust expression of HPV16 genes (> 1,000 HPV16-derived sequencing reads) were selected for further analysis (Additional file 1: Table S1). The precomputed gene expression values (fragments per kilobase of transcript per million mapped reads upper quartile, FPKM-UQ) for each tumor sample were collected from TCGA.

### Sample clustering

From the numbers of sequencing reads mapping to the HPV16 genes E2, E5, E6 and E7 [11], the relative abundances of corresponding transcripts were calculated. Based on the relative expression of E2 and/or E5, tumor samples were divided into “E2/E5-high” and “E2/E5-low” groups with > 10% and < 10% proportion of E2 + E5 among total HPV transcripts, respectively.

For immunological grouping of tumor samples, unsupervised clustering was performed based on the top 1,200 expressed genes from the Gene Ontology (GO) category “GO:0002376**—**immune system process”. Samples were clustered using correlation distance and complete linkage [15].

### Group comparisons

Differential gene expression between sample groups was calculated as the ratio of mean normalized expression values at the cBioPortal for Cancer Genomics [16, 17]. Enrichment analysis was performed with at least twofold up- or downregulated genes using Enrichr [18–20]. Tumor-infiltrating immune cells were estimated from the TCGA bulk tumor transcriptomes by deconvolution with CIBERSORTx [21]. Scores of absolute levels were calculated after batch correction (B-mode) using the LM22 signature matrix and 1000 permutations. Comparison of survival between sample groups was calculated using the cBioPortal for Cancer Genomics [16, 17]. Immunophenoscore values [22] were obtained from The Cancer Immunome Atlas (TCIA).

### Statistical analysis

The difference between groups was calculated by two-way analysis of variance and Fisher’s least significant difference test for multiple comparisons using Prism 8.4 software (GraphPad Software, San Diego, CA, USA). A p value < 0.05 was considered statistically significant.

## Results

### Delineation of tumor sample groups with different immune-related expression profiles

The CC and HNSCC tumor samples in the TCGA database were previously characterized with respect to HPV status by Ren et al. [11]. For consistency, only samples expressing HPV16, the most common high-risk HPV type [23], were selected for our analysis. Among these, only tumor samples with robust expression of HPV genes were retained. This resulted in 138 and 54 samples of CC and HNSCC tumors, respectively.

**Fig. 1:**
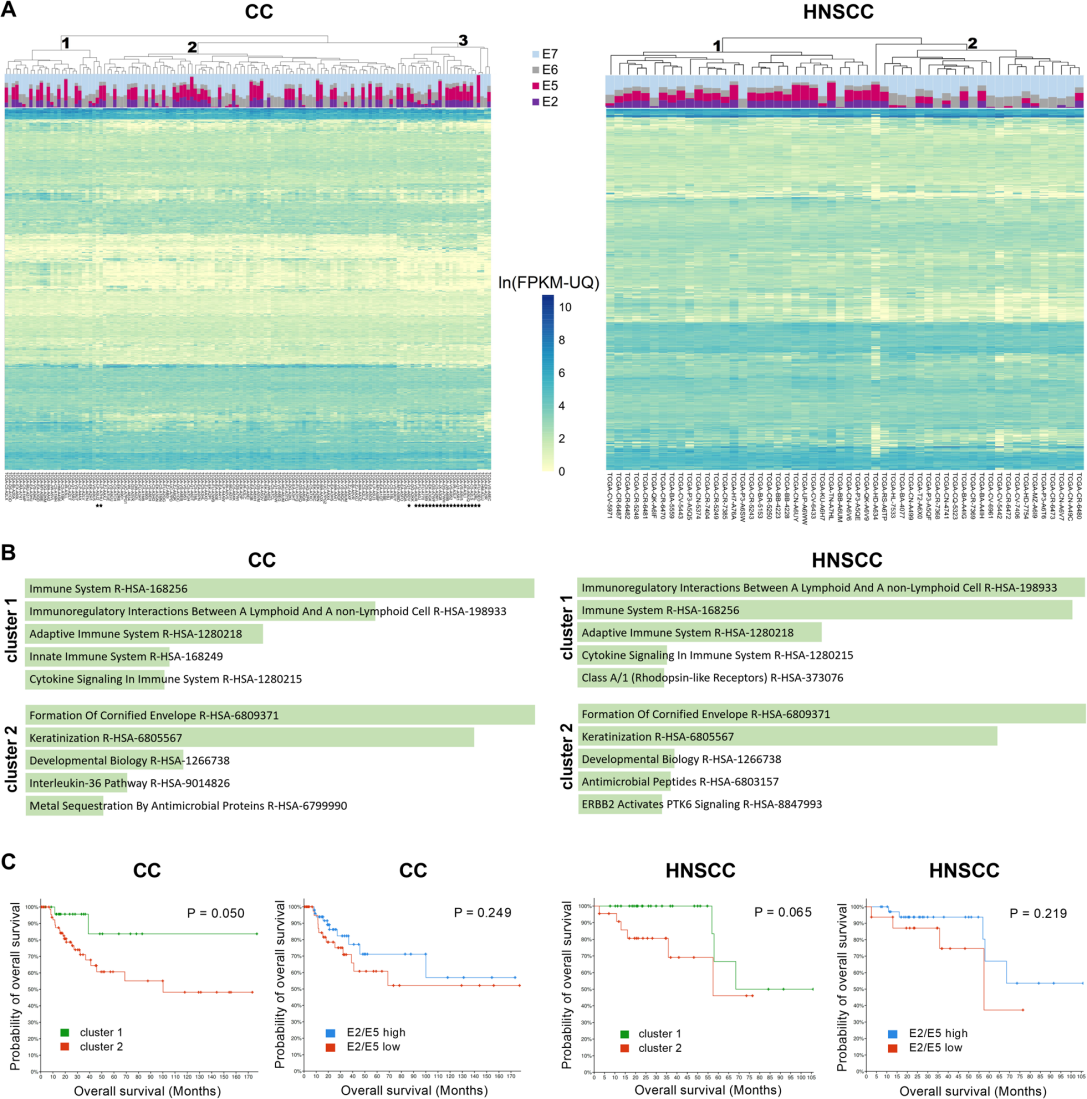
Clustering of HPV-driven tumors. **A** Samples of cervical carcinoma (CC) and head and neck squamous cell carcinoma (HNSCC) from The Cancer Genome Atlas were clustered based on the similarity of their expression profiles of immune-related genes. Relative proportions of HPV transcripts (E2, E5, E6 and E7) are shown. Cluster numbers are denoted at corresponding nodes. Nonsquamous CC samples are marked with asterisks. **B** Differences between clusters 1 and 2 were evaluated by enrichment analysis with the Enrichr tool using all genes with upregulated expression (≥ 2) in clusters 1 and 2. Reactome identifiers were sorted by p value ranking. **C** Survival analysis from cBioPortal comparing patients from clusters 1 and 2 or patients with high and low E2/E5 expression

Since the type and intensity of immune infiltration of the tumor microenvironment is a crucial factor affecting patient prognosis and treatment efficacy [24], we performed unsupervised clustering on tumors of each anatomical location based on the expression of immune-related genes (GO:0002376 “immune system process”). This revealed the presence of three and two clusters within the diversity of the CC and HNSCC samples, respectively (Fig. 1A). Cluster 3 of CC samples was predominantly composed of nonsquamous cell neoplasms (adenocarcinomas), reflecting the distinctness of global expression profiles of cervical adenocarcinomas [25]. The division of squamous cell CC samples into two clusters also reflected the clustering of their global expression profiles [26]. Nonsquamous CCs were excluded from subsequent analyses.

We then compared the expression patterns of HPV genes (E2, E5, E6 and E7) between clusters of samples. Both CC and HNSCC datasets consisted of a similar proportion of samples with negligible/low (< 10%) and high (> 10%) expression of E2/E5. In squamous CCs, both cluster 1 and cluster 2 showed a similar representation of HPV expression patterns (60% and 49% of E2/E5-high samples, respectively). In contrast, HNSCC clusters 1 and 2 consisted predominantly of E2/E5-high (93%) and E2/E5-low samples (61%), respectively.

Differential expression of all genes was used for enrichment analysis comparing samples in clusters 1 and 2 (Fig. 1B). In both tumor types, activation of immune responses was found in cluster 1, and keratinization was a major feature of cluster 2, which was associated with upregulation of oncostatin M, interleukin (IL)-36, and IL-17 signaling.

Survival analysis showed a better overall survival (OS) of patients in cluster 1, which reached statistical significance in CC (P = 0.05). Patients with high E2/E5 expression had slightly better OS than patients with low E2/E5 expression, and this difference was similar in both tumor types.

### Tumor-infiltrating leukocytes

**Fig. 2:**
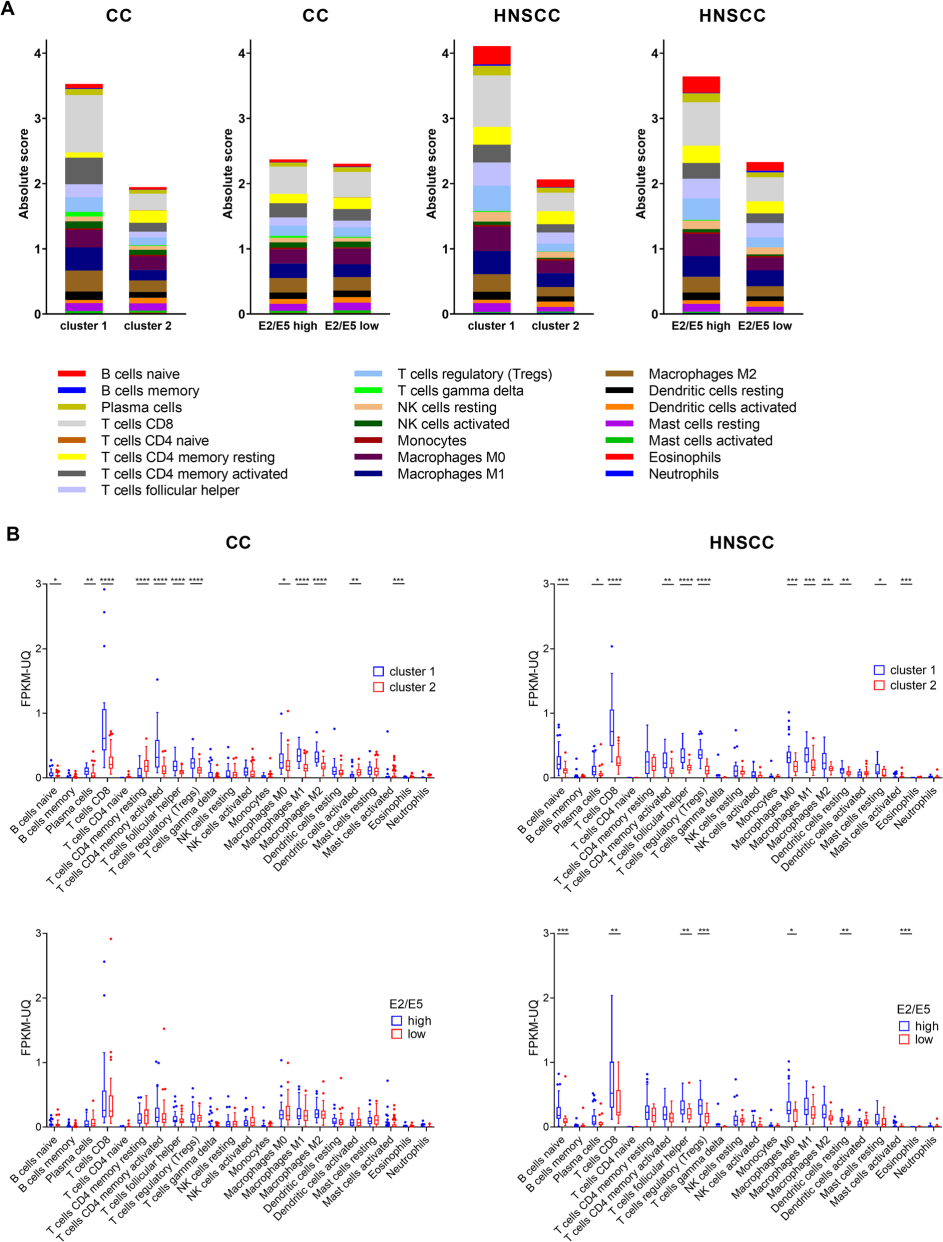
Tumor-infiltrating leukocytes. Transcriptomes of CC and HNSCC tumors were analyzed with CIBERSORTx to estimate infiltrating immune cells and compare their levels between samples from clusters 1 and 2 or groups with high and low E2/E5 expression. **A** Overview of infiltrating cell composition. **B** Statistical comparison of cell types. FPKM-UQ, fragments per kilobase of transcript per million mapped reads upper quartile; *p < 0.05, **p < 0.01, ***p < 0.001, ****p < 0.0001

To estimate the immune cell infiltration of CC and HNSCC tumors, we analyzed bulk RNA-seq data with CIBERSORTx (Fig. 2A). For both cancer types, the infiltration of cluster 1 tumors was almost twice that of cluster 2 tumors, and significant differences were observed mainly in cluster of differentiation (CD) 8^+^, CD4^+^ memory activated, follicular helper, and regulatory T cells, as well as in M1 and M2 macrophages (Fig. 2B). When comparing E2/E5-high and E2/E5-low tumors, groups of CC samples showed comparable infiltration, but in HNSCC, the samples with high E2/E5 expression were more infiltrated with immune cells. This difference was not as large as the difference between clusters 1 and 2. While remarkably higher levels of B cells and follicular helper T cells were found in HNSCC tumors, more T cells and activated natural killer (NK) cells infiltrated the CC tumors.

### Characterization of tumor subtypes

**Fig. 3:**
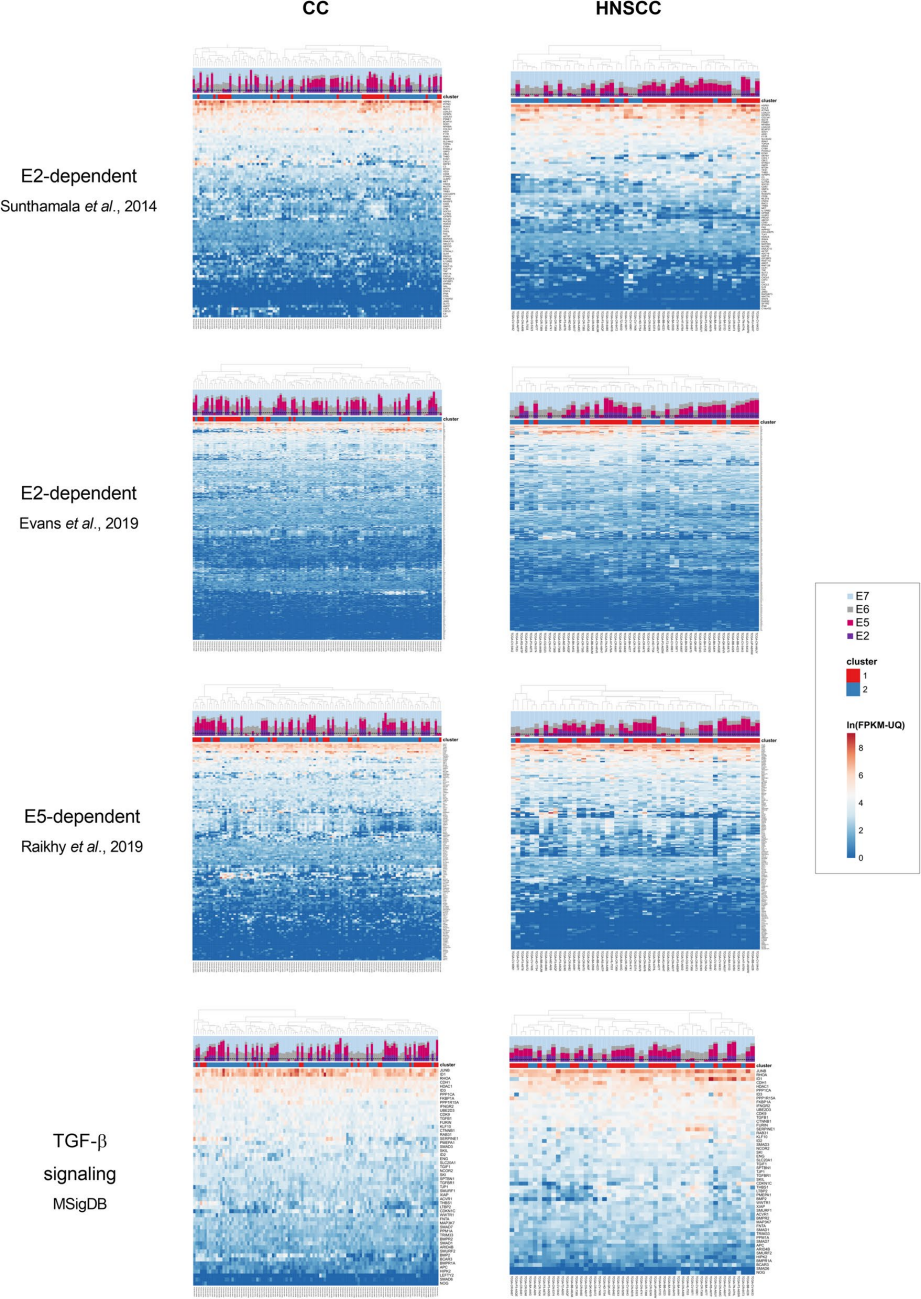
Expression of genes regulated by E2 or E5 proteins. Unsupervised clustering of HPV-expressing CC and HNSCC tumor samples was based on the expression patterns of E2-regulated, E5-regulated, and TGF-β signaling genes. Relative proportions of HPV transcripts (E2, E5, E6 and E7) are shown; the threshold that separates the E2/E5-high and E2/E5-low tumors is delineated by dashed lines

Several transcriptomic studies have analyzed the effect of HPV16 early protein expression in keratinocytes and identified upregulated and downregulated cellular genes. To further investigate the role of high E2 and E5 expression in a subset of CC and HNSCC tumors, we used the genes previously reported to be regulated by E2 [27, 28] or E5 proteins [29]. In addition, because the E5 oncoprotein has been shown to attenuate transforming growth factor (TGF)-β signaling, which is important for epithelial carcinogenesis [30, 31], we also evaluated this pathway using genes listed for TGF-β signaling in the Molecular Signatures Database (MSigDB; https://www.gsea-msigdb.org/gsea/msigdb). Unsupervised clustering did not reveal any association of the increased E2/E5 expression in CC or HNSCC samples with the expression of E2-regulated genes, E5-regulated genes or TGF-β signaling pathway genes (Fig. 3).

**Fig. 4:**
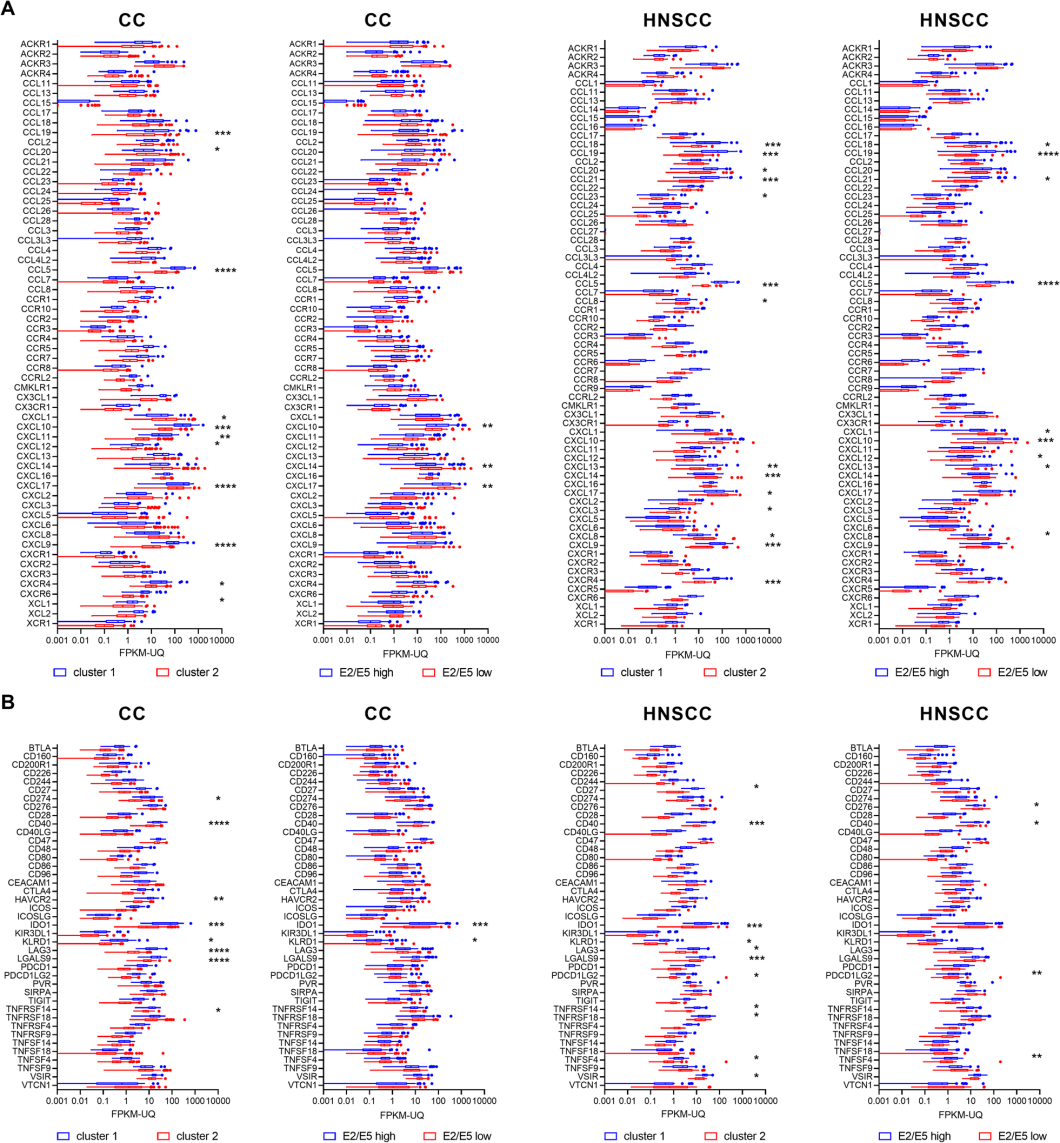
Differential gene expression. The expression of genes encoding chemokines and their receptors (**A**) or immune checkpoints (**B**) was compared between samples of cluster 1 and cluster 2 or tumors with high and low E2/E5 expression. FPKM-UQ, fragments per kilobase of transcript per million mapped reads upper quartile; *p < 0.05, **p < 0.01, ***p < 0.001, ****p < 0.0001

Next, we compared the expression of two categories of immune-related genes that are critical for the composition and functionality of the tumor microenvironment: chemokines and their receptors and immune checkpoints (Fig. 4). More genes in both categories had significantly different expression when comparing the cluster 1 and cluster 2 samples than when comparing E2/E5-high and E2/E5-low tumors. This difference was more pronounced in CC. The chemokines CCL5, CCL19, and CXCL9 and the immune checkpoints CD40, IDO1, and LGALS9 were highly upregulated in cluster 1 of both CC and HNSCC, whereas CXCL10 was dominant in cluster 1 only in CC and CCL21 only in HNCC. The expression of any chemokine or immune checkpoint was not specifically associated with the level of E2/E5 expression.

**Fig. 5:**
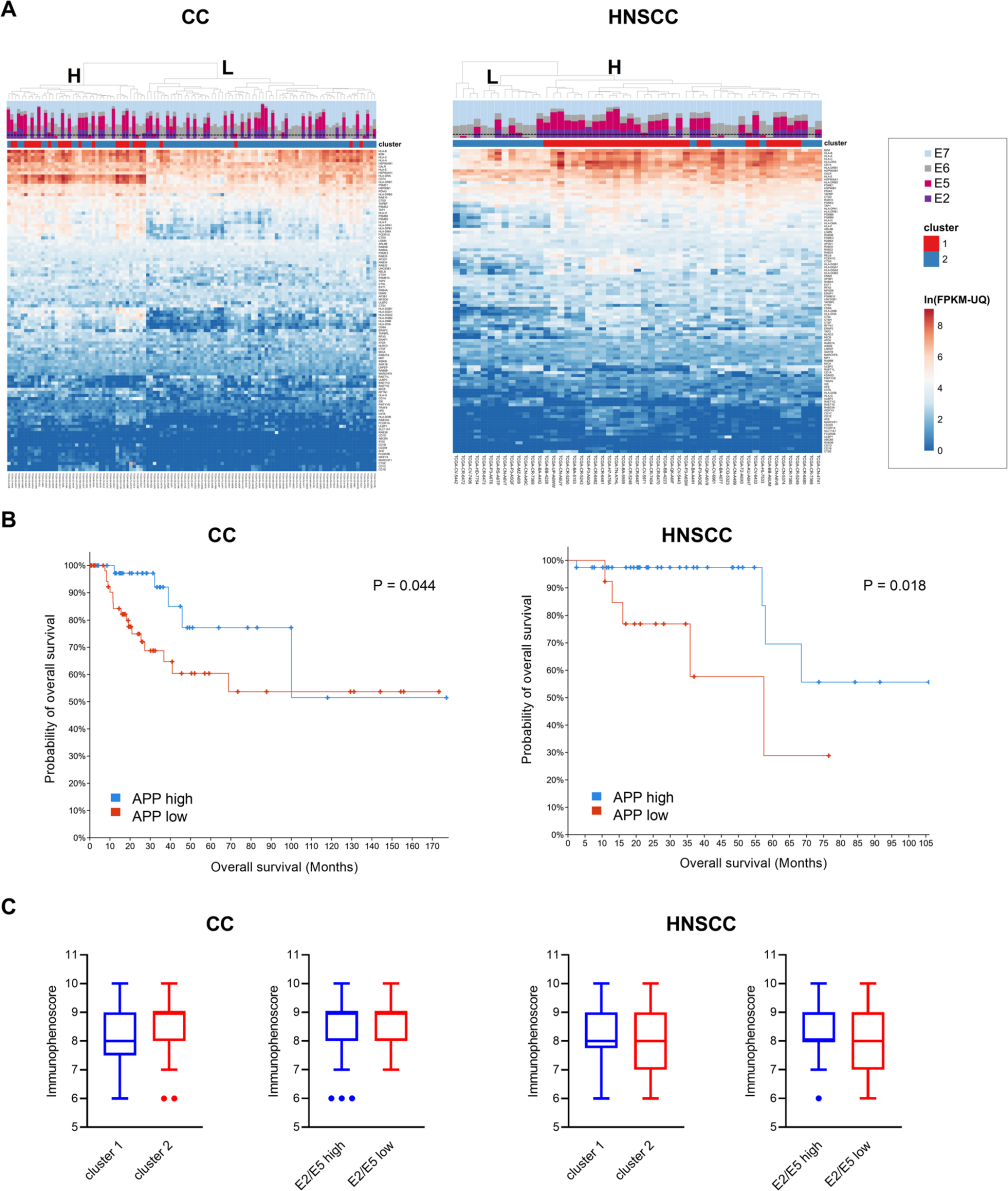
Immunogenicity of tumors. **A** Unsupervised clustering of HPV-expressing CC and HNSCC tumor samples based on the expression of antigen processing and presentation (APP) pathway components. Relative proportions of HPV transcripts (E2, E5, E6 and E7) are shown; the threshold that separates the E2/E5-high and E2/E5-low tumors is delineated by dashed lines. **B** Survival analysis compared patients from clusters with high (H) or low (L) expression of APP genes. **C** Immunophenoscore was obtained from The Cancer Immunome Atlas. Median values are indicated

Components of the antigen processing and presentation (APP) pathway are crucial for the immunogenicity of tumor cells and their recognition and elimination by adaptive immunity. Since papillomavirus proteins may affect the expression of some of these components, we selected APP genes from the GO database (GO:0019882) and evaluated their expression in CC and HNSCC samples. In both tumor types, unsupervised clustering revealed two groups of samples (designated H and L, for high and low APP expression, respectively) that were not associated with the level of E2/E5 expression (Fig. 5A). For both tumor types, the L group contained mostly samples from cluster 2, which are characterized by low immune cell infiltration. Patients from the H group of both cancer types had significantly better OS (Fig. 5B).

To characterize tumor immunogenicity, we also used the immunophenoscore (IPS), which has been shown to predict the response to immune checkpoint blockade in melanoma [22]. This parameter aggregates immune-related factors of 4 categories: (i) infiltration with CD4^+^/CD8^+^ T cells, (ii) infiltration with immunosuppressive cells (regulatory T cells and myeloid-derived suppressor cells), (iii) expression of major histocompatibility (MHC) class I, class II, and nonclassical molecules, and (iv) expression of costimulatory and coinhibitory molecules (mostly immune checkpoints). However, the comparison of IPS values obtained from TCIA did not show a significant difference even for clusters 1 and 2 (Fig. 5C).

## Discussion

Identification of molecular subtypes of tumors reveals biological differences that have important implications for cancer prognosis and treatment. In HNSCC, these studies have mostly focused on differences between HPV^+^ and HPV^−^ tumors, but heterogeneity in HPV^+^ tumors has also been investigated [32]. Two basic molecular subtypes have been identified in HPV^+^ tumors of both HNSCC [33–35] and CC [36] tumor types: (i) immune strong and (ii) highly keratinized (in terms of the reference [35]). A meta-analysis of 11 studies stratified highly keratinized HNSCC tumors into two subtypes: (i) epithelial-mesenchymal transition (EMT)-related (with high stromal score and hypoxia) and (ii) proliferation-related (with low stromal score) [37]. This classification has prognostic significance, with the best OS in patients with immune-related tumors and the worst OS in patients with EMT-related tumors. Similarly, CC patients with a strong immune response exhibited superior survival [36]. Several other bioinformatics analyses of the CC transcriptomic dataset from TCGA showed the prognostic significance of the expression of immune-related genes and/or deconvoluted levels of infiltrating immune cells [38–43].

In our study, we aimed to investigate possible differences in the expression of immune-related genes between groups of HPV^+^ tumors with alternative carcinogenesis pathways distinguished by the level of E2/E5 expression [11]. Since the E2 and E5 proteins influence the expression and function of various human proteins involved in both innate and adaptive immunity, the type of carcinogenesis may be associated with different immune characteristics that influence patient prognosis and response to immunotherapy. To improve the homogeneity of the sample groups, we focused on HPV16^+^ CC and HNSCC tumors and excluded nonsquamous tumors from the CC dataset. Unsupervised clustering identified clusters 1 with high expression of immune-related genes and clusters 2 characterized by keratinization, consistent with clustering in previous studies [33–36]. Comparisons between these clusters were used to evaluate differences between tumors with high or low expression of the E2/E5 genes.

The proportion of E2/E5-high tumors was comparable in cluster 1 and cluster 2 (60% and 49%, respectively) in CC, but it was predominant in cluster 1 (93%) and lower in cluster 2 (39%) in HNSCC. Higher expression of E2 and E5 genes (associated with lower integration) in a cluster of tumors with a high immune response was also found in another cohort of HNSCC [35]. Despite this difference in E2/E5 expression between CC and HNSCC, which was accompanied by a difference in immune cell infiltration, OS was comparably better in E2/E5-high tumors in both cancer types.

In the immune-related genes, we took a closer look at the genes that encode proteins that are critical for immune cell infiltration (chemokines and their ligands) and the efficacy of antitumor immunity (immune checkpoints and components of APP pathways). Comparison of HPV^+^ and HPV^−^ CCs has shown increased expression of APP genes and genes encoding immune checkpoints and markers of immune cells in HPV^+^ tumors [44, 45]. Since the E7 oncoprotein downregulates the expression of MHC class I genes and these genes were also upregulated in HPV^+^ tumors, the increased expression of the followed genes in HPV^+^ tumors was probably associated with higher immune cell infiltration and interferon (IFN)-γ production. Similarly, differences in the expression of immune-related genes found in our analysis between samples from clusters 1 and 2 and tumors with high and low E2/E5 expression corresponded to the levels of immune infiltrating cells, and we did not find any gene with significantly different expression between E2/E5 high and E2/E5 low tumors that could be attributed to the level of E2 or E5 expression (i.e., an immune-related gene without differential expression between clusters 1 and 2).

Downregulation of APP pathway components is a well-known mechanism of tumor immune escape that can contribute to tumor progression and lead to resistance to cancer immunotherapy, including blockade of PD-1/PD-L1 signaling [46]. As confirmed by our analysis, low expression of APP components, which is associated with worse prognosis, is common in CC and HNSCC. Therefore, approaches of cancer immunotherapy that take into account this downregulation should be applied against such tumors [47–49]. In tumors with reversible MHC class I downregulation, this expression can be restored by the induction of IFN signaling [47]. However, stimulation of IFN pathways also upregulates compensatory mechanisms that prevent autoimmune damage of tissues. In our analysis, we found increased expression of immune checkpoints in tumors with high immune cell infiltration, and we also noticed high expression of 11 leukocyte immunoglobulin-like receptor (*LILR*) genes. This family encodes both activating (LILRA1-6) and inhibitory receptors (LILRB1-5) [50]. Some LILR members bind MHC class I molecules. For example, LILRB1 binding is dependent on the expression of β-2 microglobulin and provides a ‘don’t eat me’ signal to macrophages [51]. Since LILRB1 is also expressed on NK cells and its engagement of MHC class I molecules (including nonclassical HLA-G molecules) inhibits cell lysis by NK cells, LILRB1 blockade could be used for immunotherapy of both MHC class I-proficient and MHC class I-deficient HPV-driven tumors.

In our study, we did not find an association of E2 or E5 expression with the immune characteristics of CC and HNSCC tumors or patient survival. Rather, the expression of E6 and E7 genes associated with HPV DNA integration seems to be a major viral factor affecting tumor progression and severity. When the expression of 7 HPV16 proteins (L1, L2, E1, E2, E5, E6, E7) was analyzed in CC, only E6 and E7 were significantly associated with OS, and significantly increased E6 activity was detected in the cluster of patients with a high immune response [35, 36], which is consistent with the finding that the E6 and E7 oncoproteins inhibit keratinocyte differentiation, including keratinization [52, 53]. In addition, spliced E6* isoforms, which are associated with poorer prognosis, were increased in the high keratinization cluster [35].

Although we did not find immune-related genes differentially expressed in E2/E5-high *versus* E2/E5-low tumors in this analysis of transcriptomic datasets, the level of HPV proteins may affect the level and function of some cellular immune-related proteins, which we were not able to detect in our analysis. For example, the cyclic GMP–AMP synthase (cGAS)/stimulator of interferon genes (STING) signaling pathway, that can be critical for protection against infection and in cancer immunotherapy [54] can be inhibited by several HPV proteins. While the HPV18 E7 protein can directly bind STING [55] and inhibit downstream NF-κB signaling [56], the HPV16 E7 protein destabilizes STING via the mitochondrial NOD-like receptor family member X1 (NLRX1) binding [57]. In addition, both HPV16 and HPV18 E7 proteins downregulate STING and cGAS expression by upregulating the histone methyltransferase SUV39H1 [58]. The E5 protein has also been shown to bind STING and inhibit downstream IFN signaling [59] and the E2 protein downregulates STING and IFN-κ expression [27]. Finally, the E6 protein binds and inhibits the interferon regulatory factor-3, a component of the STING pathway [60]. Suppression of STING signaling limits the effect of STING agonists in the treatment of HPV-associated tumors [59, 61, 62], but the relative contribution of individual HPV proteins and the difference between E2/E5-low and E2/E5-high tumors have not been investigated.

Finally, the E2 and E5 oncoproteins themselves could be used as targets for vaccination. Therapeutic vaccines against HPV-associated tumors are usually based only on the E7 and/or E6 oncoproteins, which are considered indispensable for maintaining the malignant transformation of cells [63]. However, the detection of specific immunity against HPV in HNSCC has shown a broad response of CD4 and CD8 T cells against viral antigens E1, E2, E4, E5, E6, E7, and L1 [64], and E1, E2 and E5 have been identified as major targets of intratumoral CD8 T cells [65, 66]. T-cell responses to E2 are also common in cervical premalignant and malignant lesions [67, 68]. Vaccination against E2 may be particularly beneficial in patients with E2/E5-high tumors. For example, a virus-based vaccine carrying conserved elements of HPV16/18/31/52/58 E1/E2/E4/E6/E7 could be applied [69].

## Conclusions

Our results suggest that while the regulation of expression of immune-related genes by the viral E2 and E5 proteins, which was found in transfected/transduced keratinocytes, is probably important for evasion of the immune system and reproduction of HPVs in nonmalignant lesions, expression of E2 and E5 is not the major determinant of immunological characteristics of tumors, especially in CC. The major role of E2 and E5 expression in carcinogenesis may be in deregulation of the E6/E7 oncogenes after E2 downregulation and/or modulation of the function of some cellular proteins.

### Supplementary Information

**Additional file 1** **Table S1** CC and HNSCC samples from TCGA used in the study.

## Data Availability

All data generated or analyzed during this study are included in this published article and its supplementary information file.

## Abbreviations

APP: Antigen processing and presentation
CC: Cervical cancer
cGAS: Cyclic GMP–AMP synthase
EMT: Epithelial-mesenchymal transition
FPKM-UQ: Fragments per kilobase of transcript per million mapped reads upper quartile
GO: Gene Ontology
HNSCC: Head and neck squamous cell carcinoma
HPV: Human papillomavirus
IL: Interleukin
IFN: Interferon
IPS: Immunophenoscore
LILR: Leukocyte immunoglobulin-like receptor
MHC: Major histocompatibility complex
MSigDB: Molecular Signatures Database
NK: Natural killer
NLRX1: NOD-like receptor family member X1
OS: Overall survival
STING: Stimulator of interferon genes
TCGA: The Cancer Genome Atlas
TCIA: The Cancer Immunome Atlas
TGF: Transforming growth factor
